# Multi‐Site Theta‐tACS Improves Memory and Language Performance and Associated Local and Remote Functional Connectivity in Mild Cognitive Impairment

**DOI:** 10.1002/cns.70707

**Published:** 2025-12-18

**Authors:** Zhiwei Guo, Yi Jiang, Jiayuan He, Ning Jiang

**Affiliations:** ^1^ National Clinical Research Center for Geriatrics, West China Biomedical Big Data Center, West China Hospital Sichuan University Chengdu China; ^2^ Institute of Rehabilitation and Imaging of Brain Function The Second Clinical Medical College of North Sichuan Medical College, Nanchong Central Hospital Nanchong China

**Keywords:** functional connectivity, mild cognitive impairment, regional homogeneity, transcranial alternating current stimulation, working memory training

## Abstract

**Background:**

While multi‐site noninvasive brain stimulation demonstrates enhanced cognitive benefits in mild cognitive impairment (MCI), the efficacy of transcranial alternating current stimulation (tACS) in this paradigm remains unestablished. The study aims were to investigate the therapeutic effects of multi‐site tACS on MCI patients and its underlying neural mechanism.

**Methods:**

A parallel, sham‐controlled trial was conducted with 60 MCI participants assigned to multi‐site tACS (prefrontal gyrus and precuneus), single‐site tACS (precuneus), or sham groups. All participants underwent 20 days of 7 Hz tACS concurrent with working memory training. Cognitive assessments and fMRI examinations were performed pre‐ and post‐ intervention. Local and remote functional connectivity changes were evaluated using regional homogeneity (Reho) and resting‐state network (RSN) analyses, respectively.

**Results:**

Multi‐site tACS demonstrated superior cognitive improvements, particularly in verbal fluency (*p* = 0.006) and recognition memory (*p* = 0.025), compared to sham stimulation. Significant ReHo changes were observed in the superior frontal gyrus (SFG) and superior temporal gyrus (STG) exclusively in the multi‐site group (*p* < 0.05). Additionally, multi‐site tACS induced broader functional connectivity alterations in the default mode network (DMN), executive control network (ECN), and left frontoparietal network (FPN). Correlations were found between the Reho changes in SFG and STG and the score changes of immediate memory (*r* = 0.367, *p* = 0.039) and language naming (*r* = 0.371, *p* = 0.037), respectively. Functional connectivity changes in the right inferior parietal lobe were also significantly correlated with the score changes of language naming (*r* = −0.374, *p* = 0.035). Moreover, more functional connectivity changes between the prefrontal gyrus stimulation site and the salience network and DMN were also detected in the multi‐site group relative to the single‐site group.

**Conclusions:**

Multi‐site tACS is more effective than single‐site tACS in enhancing cognitive functions and modulating cognition‐related brain networks in MCI patients. These superior neuromodulatory effects of multi‐site tACS may be attributed to its capacity to modulate functional networks across the prefrontal gyrus more extensively.

## Graphical abstract

Multi‐site tACS over both the prefrontal gyrus and precuneus yields superior cognitive improvement in MCI, particularly in recognition memory and verbal fluency, by modulating more cognition‐related functional networks—an effect that may be underpinned by enhanced stimulation of the frontal lobe.

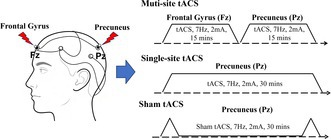

## 1 Introduction

Mild cognitive impairment (MCI) is considered a transitional stage between normal aging and Alzheimer's disease (AD), characterized by early stage of memory loss or other cognitive ability loss exceeding expected age and education related changes [1, 2]. Approximately 15% MCI patients progress to dementia within 2 years [3], with about 1/3 developing dementia due to AD within 5 years [4]. While current pharmacological treatments may slow cognitive decline of AD, they are not curative and often present significant side effects and are costly [5]. Consequently, early intervention during MCI stage has become particularly crucial for delaying dementia onset of AD [6]. MCI has also been considered the critical time window for early prediction of conversion to AD [7] and early prevention against dementia. Given the limited efficacy of clinical pharmacological interventions in treating AD and MCI, nonpharmacological treatments such as non‐invasive brain stimulation technique appear to be promising [8, 9].

Non‐invasive brain stimulation techniques, including repetitive transcranial magnetic stimulation (rTMS), transcranial direct current stimulation (tDCS), and transcranial alternating current stimulation (tACS), have been proved to be effective on improving cognitive function of MCI and AD [8, 9, 10, 11]. Notably, multi‐site NIBS intervention demonstrates enhanced efficacy compared to single‐site stimulation [12, 13]. Multi‐site NIBS stimulation has become a current research direction for exploring optimal treatment parameters and improving therapeutic efficacy. Moreover, in terms of disease mechanisms, AD is considered a disconnection syndrome, characterized by the disrupted structural and functional connectivity between various brain regions and networks [14, 15, 16, 17]. This may suggest that there should be multiple brain regions related to the disease and treatment efficacy. Multi‐site NIBS treatment is a reasonable choice and could realize better efficacy. A circuit‐based neuromodulation study has confirmed the theoretical basis for the selection of multi‐site approaches and demonstrated that multi‐site rTMS therapy can further improve memory function of MCI patients and repair damaged memory functional connectivity circuit [18]. Another study using dual‐targeted rTMS reported superior effects of dual‐targeted rTMS on more cognitive functions, including overall cognition, memory function, and executive function, in MCI patients after 4 weeks of therapy [19]. Similar results were also observed in a tDCS study using multi‐site anodal tDCS combined with cognitive stimulation, in which multi‐site tDCS could result in significantly greater changes in cognitive performance of AD patients than sham [20]. While multi‐site rTMS and tDCS have shown promise in MCI treatment, the potential of multi‐site tACS remains unexplored. Besides, the neuromodulation mechanisms are still poorly understood.

Numerous neuroimaging meta‐analyses have demonstrated aberrant local functional connectivity, resting‐state functional connectivity, cerebral blood flow, and structural changes in MCI and AD patients relative to healthy elderly people [21, 22, 23, 24]. The locations of these consistent abnormal changes are predominantly associated with cognition‐related resting‐state networks (RSNs) involving the default mode network (DMN), salience network (SN), executive control network (ECN), and frontoparietal network (FPN) [22, 25, 26, 27]. Besides, the functional connectivity of these networks could be regulated by rTMS [28, 29], tDCS [30], tACS [31], physical exercise [32, 33], and acupuncture [34]. These networks have also shown significant correlation with the clinical efficacy of interventions like physical exercise [32] and rTMS intervention [28]. These networks demonstrate responsiveness to various interventions, suggesting their utility as biomarkers for treatment efficacy assessment.

Therefore, considering these issues, the aim of this study was to explore whether multi‐site tACS yields superior cognitive benefits in MCI patients compared to single‐site stimulation, while examining associated changes in local and remote functional connectivity to evaluate the underlying neural mechanism. The selection of tACS for this study was based on three considerations: (1) comparable efficacy to other NIBS methods; (2) greater portability and cost‐effectiveness than rTMS; and (3) evidence suggesting superior cognitive benefits compared to tDCS in MCI patients [35].

## 2 Materials and Methods

### 2.1 Participants

Participants were recruited from the local community through advertisements posted on local bulletin boards. A total of 60 MCI participants provided written informed consent after being fully briefed on the study procedures, which were approved by the Ethics Committee of the Second Clinical Medical College of North Sichuan Medical College and the Ethics Committee on Biomedical Research, West China Hospital of Sichuan University, in accordance with the Declaration of Helsinki.

All MCI participants were included according to the following inclusion criteria: (1) aged 55–80 years; (2) had relevant symptoms of cognitive impairment, as reported by the patient or their family members or confirmed by a clinical physician; (3) had impairment in one or more cognitive domains, confirmed by cognitive tests; (4) a Clinical Dementia Rating (CDR) score of 0.5; (5) maintained general independent living abilities, with mild impairment in complex instrumental activities of daily living; and (6) test scores not meeting the diagnostic criteria for dementia. Exclusion criteria were: (1) history of neurological or psychiatric diseases (e.g., stroke, epilepsy, Parkinson's disease, traumatic brain injury) that could lead to cognitive decline; (2) congenital cognitive or mental retardation; (3) systematic diseases (e.g., syphilis, thyroid dysfunction, anthracemia, severe anemia, or HIV) that may cause cognitive impairment; (4) history of addiction or treatments that could interfere with cognitive abilities; or (5) inability to complete neuropsychological assessments or contraindication for MRI and rTMS.

### 2.2 Stimuli and Procedures

#### 2.2.1 Study Design

**FIGURE 1:**
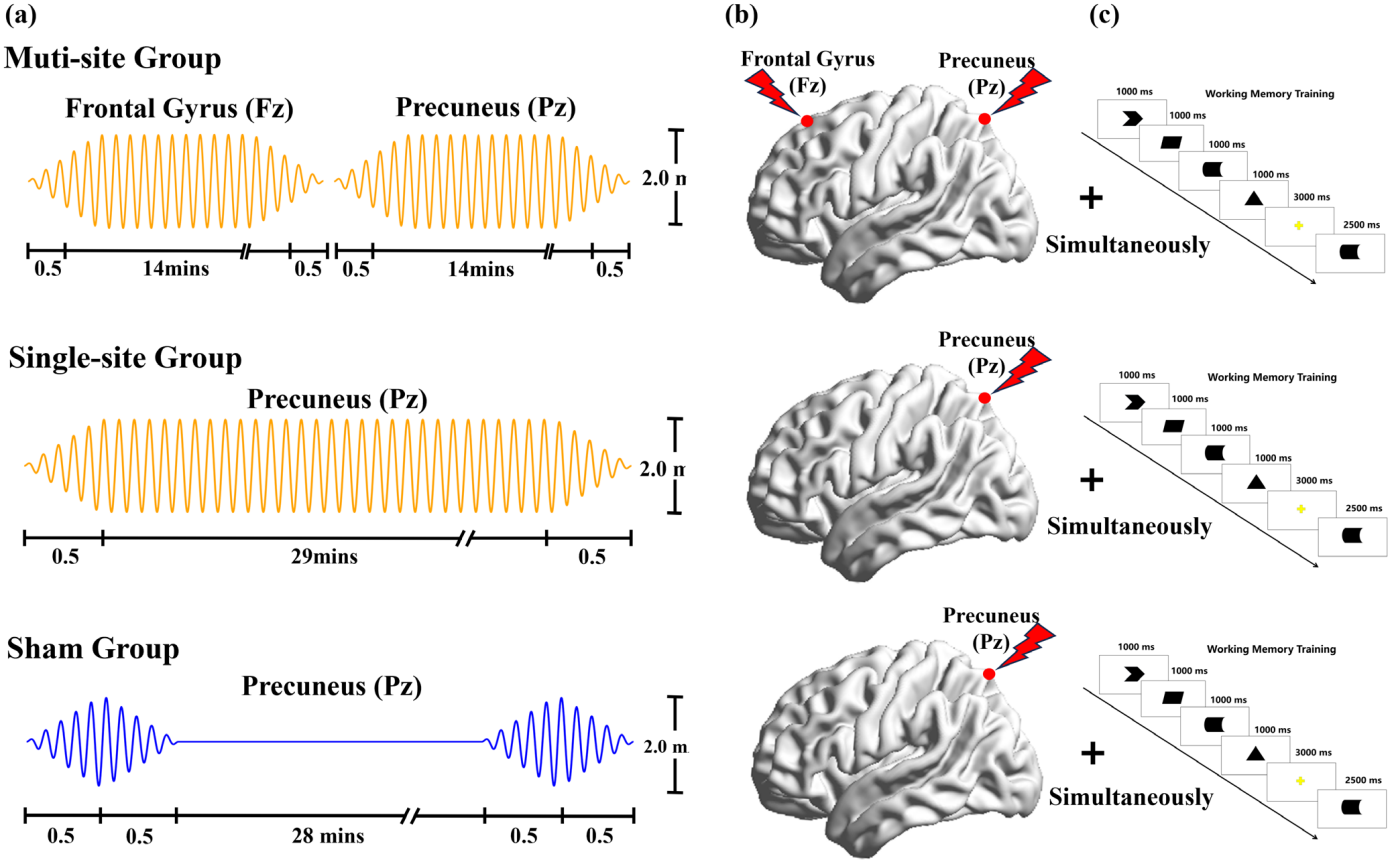
Flow chart of tACS intervention and working memory training in each group. (a) Timeline of the tACS protocol. (b) Stimulation sites for each group. (c) Working memory training task. tACS and working memory training were administered concurrently.

We conducted parallel, sham‐controlled experiments over 20 consecutive days, combining high‐definition tACS (HD‐tACS) with working memory training. MCI participants were assigned to one of three intervention groups: multi‐site tACS (*n* = 20), single‐site tACS (*n* = 20), or sham tACS (*n* = 20). The multi‐site group received sequential tACS stimulation targeting the frontal gyrus (Fz) and precuneus (Pz), with each brain region receiving 15 min of stimulation. The single‐site group received tACS only over the precuneus (Pz) for a duration of 30 min. The sham group received the same configuration as the single‐site group, but without actual tACS stimulation. All MCI participants in the three groups received synchronous working memory training during the tACS intervention period. Figure 1 illustrates the tACS stimulation procedure (Figure 1a) and stimulation sites (Figure 1b) for each experimental group.

#### 2.2.2 tACS Intervention Protocol

The tACS was delivered through a 5‐channel high‐definition transcranial electrical stimulator (Volcan VTS‐801‐D, Nanjing, China). Participants were fitted with a neoprene head cap with 64‐channel electrode positions according to the 10‐10 international standard system. Five plastic holder clips were used to secure the electrodes into the corresponding electrode positions on the cap. Ag/AgCl ring electrodes were then inserted into the clips and filled with conductive gel. For the frontal gyrus stimulation, the electrodes were placed at AFz, F1, F2, FCz, and Fz, with the anode electrode at Fz. For the precuneus stimulation, the electrodes were placed at CPz, P1, P2, POz, and Pz, with the anode electrode at Pz. During stimulation, a bipolar sinusoidal alternating current was applied at 7 Hz for each target region. The current was ramped up to 2 mA over 30 s, maintained at full strength for 14 min (multi‐site group) or 29 min (single‐site group), and then gradually decreased to 0 mA over 30 s. In the sham condition, the current was ramped up to 2 mA in the first and last minute of each session, then immediately ramped back down to 0 mA. Each participant received 20 days of continuous tACS intervention.

#### 2.2.3 Working Memory Training

The working memory training was provided by using a delayed sample‐matching paradigm. Before starting the training task, participants underwent operational training to familiarize themselves with the procedures. During the training, participants were instructed to sit in a quiet room with dim lighting while receiving synchronous tACS stimulation. The training paradigm was presented on a computer screen.

The paradigm used a series of two‐dimensional black and white geometric images to eliminate any potential influence related to image attributes such as color or familiarity. Each trial began with a red “+” cue in the center of the screen for 1.5 s, reminding participants to direct their visual attention to the center of the screen as the trial commenced. Next, four different images randomly appeared sequentially in the center of the screen, with each image displayed for 1 s. Following this, a yellow “+” symbol cue appeared in the center of the screen for 3 s, signaling participants to prepare for recall. A randomly chosen detection image then appeared for 2.5 s, and participants had to decide if this image had appeared among the previous four. If it had, participants were instructed to press the “Space” key with their left hand; otherwise, they were instructed to press the “Enter” key with their right hand. After each trial, the word “Rest” appeared in the center of the screen for 4 s, indicating a brief pause. Each session consisted of 20 trials, as shown in Figure 1c.

The tACS intervention and working memory training were administered simultaneously. To ensure that the working memory training was delivered synchronously during the tACS intervention, the training task was manually started and stopped at the beginning and end of the stimulation. However, this manual operation could not achieve precise time locking.

#### 2.2.4 Clinical Outcome Measures

Comprehensive clinical and neuropsychological assessments were conducted at baseline and immediately following the completion of 20 days of tACS intervention. These assessments included: (1) general cognitive performance, measured by the Mini‐Mental State Examination (MMSE) and the Huashan version of the Montreal Cognitive Assessment (MoCA); (2) memory function, assessed by the Auditory Verbal Learning Test (AVLT), including immediate recall (AVLT‐I), delayed recall (AVLT‐DR), and recognition (AVLT‐R); (3) language function, measured by the Boston Naming Test (BNT) and the Animal Semantic Fluency Test (AFT); and (4) executive function, evaluated using the Shape Trails Test (STT), which comprises parts A (STT‐A) and B (STT‐A). Pre‐ and post‐intervention changes in these cognitive scores were calculated to evaluate the efficacy of tACS intervention.

#### 2.2.5 MRI Data Acquisition

Each MCI participant underwent an MRI examination using a Philips Ingenia CX 3.0 T scanner at baseline and immediately after 20 days of tACS treatment session. High‐resolution T1‐weighted anatomical imaging and resting‐state functional magnetic resonance imaging (rs‐fMRI) were performed. The T1‐weighted anatomical images were acquired along the sagittal plane with the following parameters: repetition time/echo (TR/TE) = 6.67/3.02 ms, field of view = 240 × 240.0 mm^2^, flip angle = 8°, acquisition matrix = 240 × 240, voxel size = 1.0 × 1.0 × 1.0 mm^3^, slices = 170. During the fMRI scan, patients were instructed to stay awake, relax with their eyes closed and remain motionless as possible. The rs‐fMRI images were acquired with the following parameters: TR/TE = 2, 000/30 ms, field of view = 240.0 × 240.0 mm^2^, flip angle = 90°, matrix = 64 × 64, voxel size = 3.75 × 3.75 × 3.75 mm^3^, and 36 axial slices. A total of 245 volumes were continuously obtained for each scan.

### 2.3 Functional Image Analysis

#### 2.3.1 Image Preprocessing

The preprocessing of the rs‐fMRI data was carried out using the Data Processing and Analysis of Brain Imaging (DPABI V7.0) toolbox [36]. Prior to preprocessing, the first 10 volumes of the fMRI datasets were discarded to eliminate magnetization equilibrium effects and to allow for the adaptation phase of the participants. Subsequently, slice‐timing, head motion realignment, spatial normalization, and spatial smoothing steps were performed. Nuisance covariates, including 24 head motion parameters, global mean signal, white matter signal, and cerebrospinal fluid signal, were regressed out. Finally, linear detrending and temporal filtering at a frequency band of 0.01–0.08 Hz was applied. After preprocessing, participants with a maximum head translation greater than 3 mm or a maximum rotation of 2° were excluded from further analysis.

#### 2.3.2 Reho Calculation

Whole brain regional homogeneity (Reho) analysis was conducted for each MCI participant using DPABI toolbox [36]. Briefly, the Kendall's coefficient of concordance (KCC) of the time series of each voxel with the time series of its 26 nearest neighboring voxels was calculated. A voxel‐wise whole‐brain Reho map was generated for each participant. To reduce global variability across participants, the Reho value of each voxel was standardized by dividing it by the global mean Reho for each individual. Finally, the standardized Reho maps of all participants were spatially smoothed with an 8 mm full width at half maximum (FWHM) Gaussian kernel.

#### 2.3.3 Resting State Functional Network Analysis

Group‐level independent component analysis (ICA) was performed by using the GIFT v4.0b toolbox (https://trendscenter.org/software/gift/) to explore the resting‐state functional networks. Both baseline and post‐tACS datasets were used for this analysis. Two data reduction steps were carried out by using principal component analysis (PCA). The minimum description length (MDL) criterion was employed to automatically estimate the number of independent components (ICs) to retain in the subsequent ICA stage. Then, the infomax algorithm [37] was applied to decompose the reduced data from all patients into 33 estimated ICs. This calculation process was repeated multiple times for estimation of the stability [38]. Participant‐specific time courses and spatial maps were obtained from the spatiotemporal regression back reconstruction approach [39], and the results were transformed to *z* scores.

Cognitive‐related RSNs were selected for further analysis based on previously reported RSNs in fMRI studies. Components were identified through visual observation. These RSNs were selected and extracted from the ICs based on their anatomical and functional properties to identify possible neuromodulation mechanisms of tACS to MCI patients.

#### 2.3.4 Functional Connectivity Between Stimulation Sites and RSNs

To further understand the modulation of tACS over different stimulation sites to cognition‐related functional networks, the functional connectivity between the time series of each stimulation site and each RSN was calculated. A previous study on functional connectivity using EEG electrode positions as seeds has reported the MNI coordinates corresponding to each electrode of 10‐10 EEG systems [40]. Based on this study, the MNI coordinate (2, 32, 54) and (4, −64, 58) were defined as the peak coordinates of the voxel at Fz and Pz stimulation sites, respectively. The average time signal of the peak voxel and its 26 nearest neighboring voxels was calculated and used as the representative signal for each stimulation site. The time signal of each RSN was extracted from the time courses file obtained from the ICA analysis. Linear detrending and temporal filtering (0.01–0.08 Hz) were applied to these signals. Nuisance covariates, including 24 head motion parameters, global mean signal, white matter signal, and cerebrospinal fluid signal, were regressed out. After preprocessing, the Pearson correlation coefficient was calculated between the time signals of stimulation sites and cognition‐related RSNs. These coefficients were then transformed to *z* scores using Fisher's *z* transformation. Finally, a correlation matrix was obtained for each MCI participant.

### 2.4 Statistical Analysis

Comparisons of demographic information and neurocognitive scores were conducted by using IBM Statistical Package for Social Sciences (SPSS) v23.0. Paired *t*‐tests were used to test the significant cognitive improvements after tACS intervention in each group. Changes in cognitive scores post‐tACS intervention were calculated for each group. Group differences in cognitive score changes among three groups were examined with one‐way ANOVA or nonparametric test (Kruskal–Wallis), depending on the normality and homogeneity of the data. Post hoc pairwise comparisons were performed using *t*‐tests or nonparametric tests (Mann–Whitney) for multiple comparisons if the comparison among the three groups yielded significant results (*p* < 0.05).

Statistical analysis of Reho and RSNs was performed using the SPM 12 software package. Multi‐factor ANOVA was applied to explore the group, time, and group × time interaction effects. Paired *t*‐test was used to investigate the changes in functional connectivity following tACS intervention relative to baseline in each group. Functional connectivity among the time signals of stimulation sites and RSNs was compared between groups. Comparison of the correlation matrix between baseline and post‐tACS was also conducted for each group. Information on the brain regions showing significant functional connectivity differences among groups in ANOVA analysis was extracted for further correlation analysis with cognitive improvement scores. Finally, Spearman correlation coefficients were computed to assess the relationship between cognitive score changes after tACS intervention and Reho/functional connectivity changes.

## 3 Results

### 3.1 Participant Demographics

Forty‐three MCI participants completed 20 days of tACS intervention, consisting of 15 MCI in the multi‐site group, 14 in the single‐site group, and 14 in the sham group. All participants completed baseline and post‐treatment cognitive assessments. Participants who were excluded from the analysis did not provide complete data due to reasons such as withdrawal from treatment midway, unwilling to undergo post‐treatment assessments, or other factors. The demographic characteristics and baseline neurocognitive performance of the included MCI participants are summarized in Table 1. The single‐site group demonstrated significantly higher baseline scores in both AVLT‐DR and AVLT‐R than the multi‐site group (*t* = 2.446, *p* = 0.021; *z* = 2.483, *p* = 0.013). Furthermore, the sham group also showed a significantly higher baseline AVLT‐R score compared to the multi‐site group (*t* = 2.449, *p* = 0.021). After preprocessing and screening the pre‐ and post‐treatment MRI images of these MCI participants, a total of 32 subjects ultimately met the image quality requirements and were included in the MRI data analysis. Among them, 10 were from the multi‐site group, 13 from the single‐site group, and 9 from the sham group.

**TABLE 1:** Demographic characteristics and baseline neurocognitive scores of included MCI participants.

	Multi‐site (*n* = 15)	Single‐site (*n* = 14)	Sham (*n* = 14)
Age^a^	71.07 ± 6.43	67.00 ± 6.37	69.64 ± 5.06
Gender (M/F)^b^	9/6	4/10	4/10
Education^a^	7.07 ± 4.70	7.57 ± 3.41	7.08 ± 3.88
MMSE^a^	25.13 ± 2.53	25.71 ± 2.55	25.21 ± 2.42
MoCA^a^	18.07 ± 4.57	18.57 ± 3.34	18.29 ± 4.55
AVLT‐I^c^	3.00 ± 1.85	4.00 ± 1.88	2.93 ± 1.82
AVLT‐DR^c^	2.27 ± 1.62	3.86 ± 1.88^#^	2.64 ± 1.50
AVLT‐R^c^	17.00 ± 2.75	19.64 ± 2.50^#^	19.14 ± 1.83*
BNT^a^	18.33 ± 3.94	17.29 ± 3.20	17.14 ± 3.92
AFT^a^	10.67 ± 3.85	11.64 ± 2.71	11.71 ± 3.24
STT‐A^c^	127.33 ± 70.99	125.93 ± 61.78	141.07 ± 65.90
STT‐B^a^	261.93 ± 77.59	283.93 ± 117.99	298.93 ± 104.73

Abbreviations: AFT, Animal Verbal Fluency Test; AVLT, auditory verbal learning test‐immediate recall; AVLT‐DR, AVLT delayed recall; AVLT‐I, AVLT immediate recall; AVLT‐R, AVLT recognition; BNT, Boston Naming Test; F, female; M, male; MMSE, Mini‐Mental State Examination; MoCA, Montreal Cognitive Assessment; STT‐A, Shape Trails Test Part A; STT‐B, Shape Trails Test Part B.

^a^ Independent samples *t*‐test.

^b^ Chi‐squared test.

^c^ Mann–Whitney *U* test.

* Significant difference between multi‐site and sham groups.

^#^ Significant difference between multi‐site and single‐site groups.

### 3.2 Effects of tACS on Cognitive Performance

**FIGURE 2:**
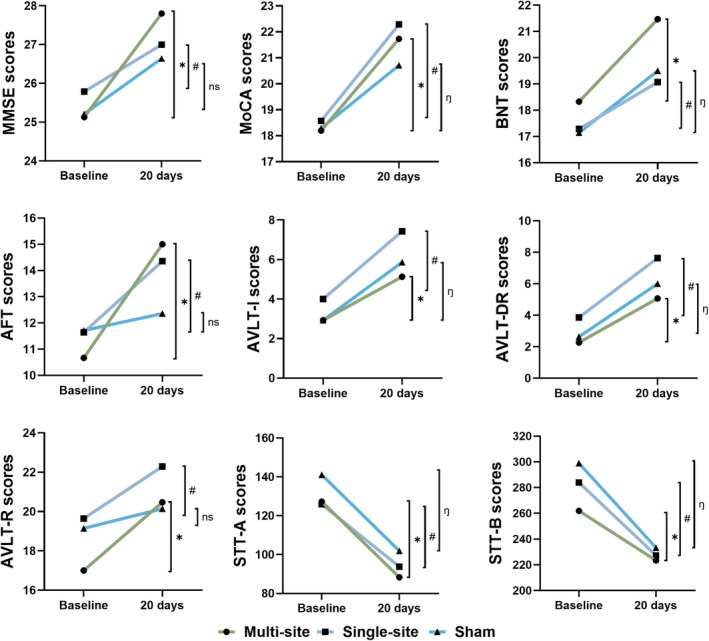
Cognitive performance at baseline and immediately after 20 days of tACS intervention. *Significant changes after multi‐site tACS intervention; ^#^significant changes after single‐site tACS intervention; η, significant changes after sham tACS intervention; ns, no significant changes.

Figure 2 illustrates the cognitive performance of each group at baseline and immediately after 20 days of tACS intervention. It is evident that all cognitive scores improved progressively with the tACS treatment. A comparison between baseline and post‐treatment cognitive scores showed significant improvements (*p* < 0.05) in all cognitive functions for both multi‐site and single‐site groups. However, the sham group only exhibited significant changes in the MoCA, BNT, AVLT‐I, AVLT‐DR, STT‐A, and STT‐B scores.

**FIGURE 3:**
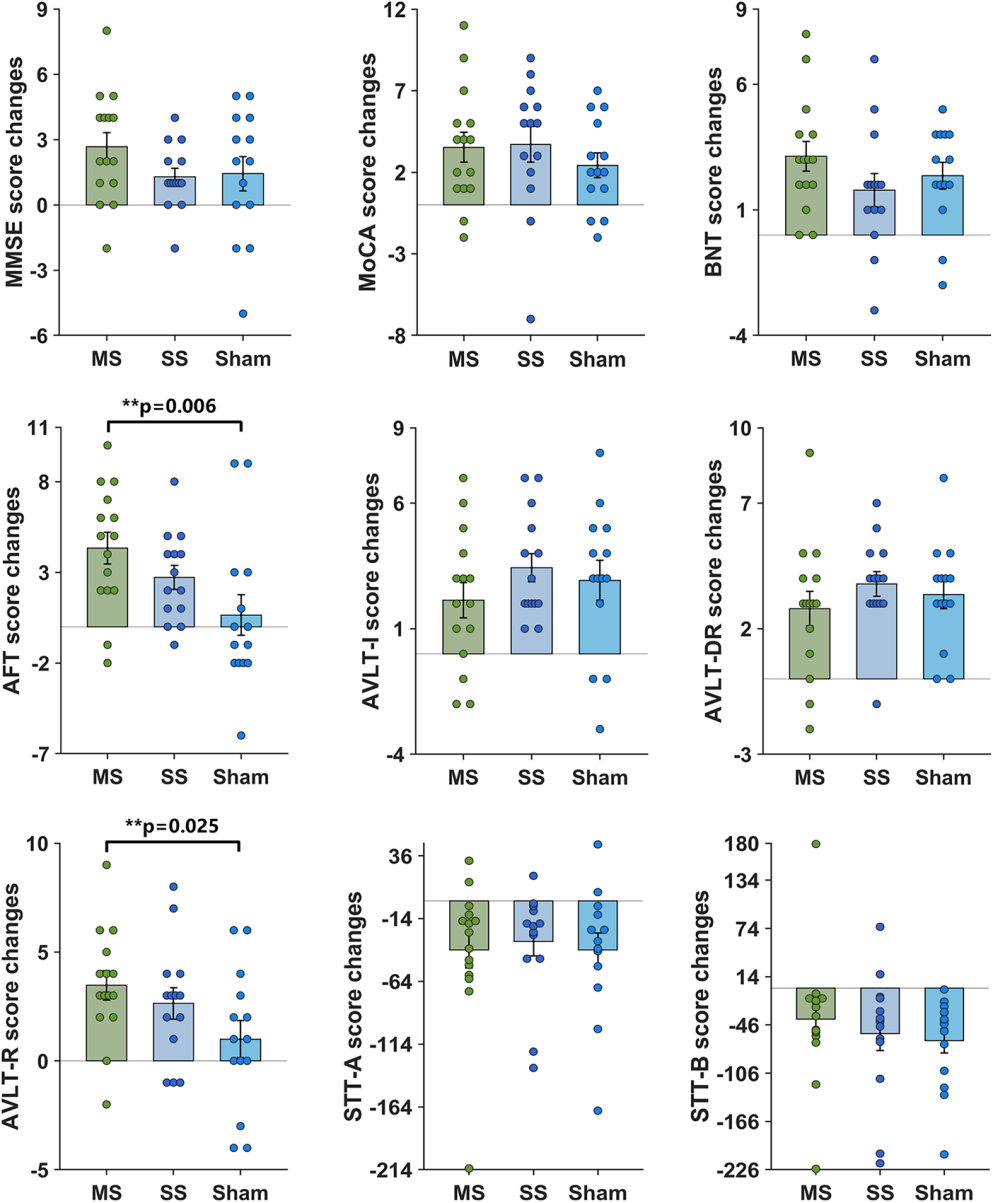
Comparison of the cognitive score changes after 20 days of tACS therapy showed significant differences in the changes of AFT and AVLT‐R between the multi‐site group and the sham group. MS, multi‐site; SS, single‐site.

To further evaluate the therapeutic advantages of multi‐site tACS, a comparison of cognitive score changes among the three groups was also conducted. ANOVA analysis showed significant group difference in the AFT score changes among the three groups (*F* = 4.23, *p* = 0.022). Post hoc analysis showed a significantly greater increase in AFT (*p* = 0.006) and AVLT‐R (*p* = 0.025) scores after 20 days of tACS in the multi‐site group compared to the sham group. No significant differences were observed between the multi‐site and single‐site groups across the cognitive scales. The results are shown in Figure 3.

### 3.3 Effects of tACS on Local Functional Connectivity in MCI Patients

**FIGURE 4:**
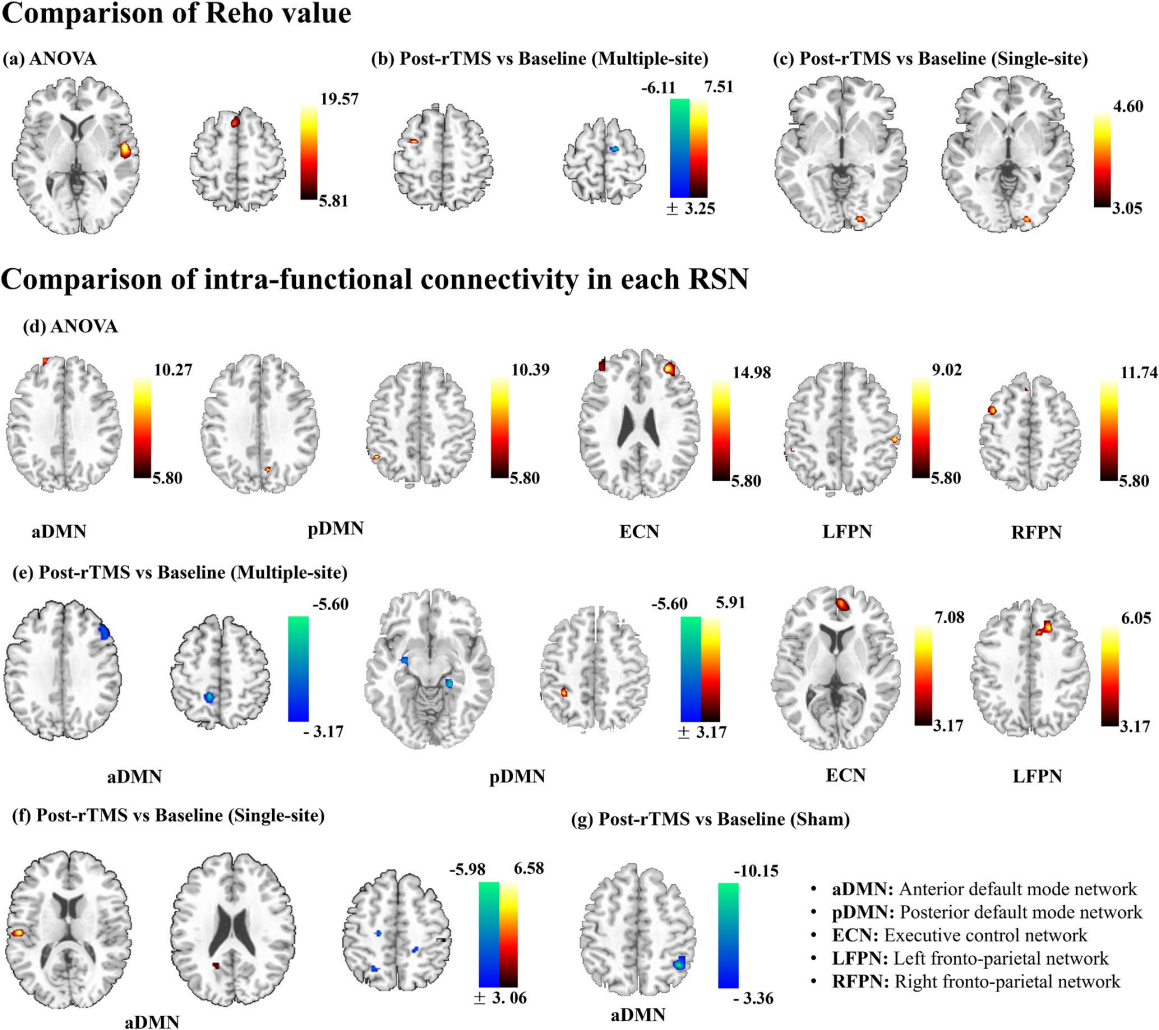
Comparison of the Reho (a), functional connectivity of each RSN (d) among multi‐site, single‐site, and sham groups, and comparisons between baseline (b, c) and post‐tACS (e, f, g).

The results of the ANOVA and post hoc analysis of Reho images among the three groups are shown in Figure 4 and Table S1 (*p* < 0.005, uncorrected). Significant main group effects were identified in the left superior temporal gyrus and bilateral superior frontal gyrus (Figure 4a). No significant time effects and group × time interaction effects were observed in the comparison. Further post hoc analysis revealed significant changes in Reho only in the multi‐site and single‐site groups after tACS treatment. In the multi‐site group, significant increases in Reho were observed in the right middle frontal gyrus, while decreases were found in the left superior frontal gyrus (Figure 4b) (*p* < 0.005, uncorrected). Conversely, the single‐site group showed an increase in Reho in the left inferior occipital lobe (Figure 4c) (*p* < 0.005, uncorrected). No significant Reho changes were observed in the sham group after tACS intervention.

### 3.4 Effects of tACS on Functional Connectivity of RSNs in MCI Patients

To assess whether modulation patterns differ with different stimulation modalities, we analyzed the functional connectivity difference and alteration within cognition‐related RSNs. Significant main group effects were found within the anterior part of the DMN (aDMN), posterior part of the DMN (pDMN), ECN, left FPN (LFPN), and right FPN (RFPN), primarily in the right superior frontal gyrus, left precuneus, bilateral inferior parietal lobe, and bilateral middle frontal gyrus (Figure 4d; Table S2; *p* < 0.005, uncorrected). No significant time effects and group × time interaction effects were observed in the comparison. Compared to baseline, we observed significantly decreased functional connectivity in the right precuneus and left middle frontal gyrus within aDMN, as well as in the right hippocampus and left parahippocampal gyrus within pDMN. Additionally, significantly increased functional connectivity was observed in the right superior frontal gyrus within aDMN, right superior temporal gyrus, and right inferior parietal lobe within pDMN, left superior medial frontal gyrus and left superior parietal lobe within ECN, and left superior frontal gyrus in the LFPN after tACS treatment in the multi‐site group (Figure 4e; Table S2; *p* < 0.005, uncorrected).

For the single‐site group, significant increases in functional connectivity were detected in the right precuneus, left inferior parietal lobe, and right superior temporal gyrus within aDMN after tACS intervention (Figure 4f; Table S2; *p* < 0.005, uncorrected). Furthermore, the sham group exhibited significant decreases in functional connectivity in the left inferior parietal lobe (Figure 4g; Table S2; *p* < 0.005, uncorrected).

### 3.5 Modulation of Stimulation Over Different Sites to RSNs

Regarding functional connectivity between stimulation sites and RSNs, inter‐group differences were observed in changes in functional connectivity between Fz and SN (*p* = 0.019), as well as between Fz and aDMN (*p* = 0.012) in the multi‐site group and single‐site groups. A significant inter‐group difference in changes in functional connectivity between Fz and SN (*p* = 0.023) was also found between the single‐site group and sham groups. No significant inter‐group differences were observed in changes in functional connectivity between Pz and RSNs.

**FIGURE 5:**
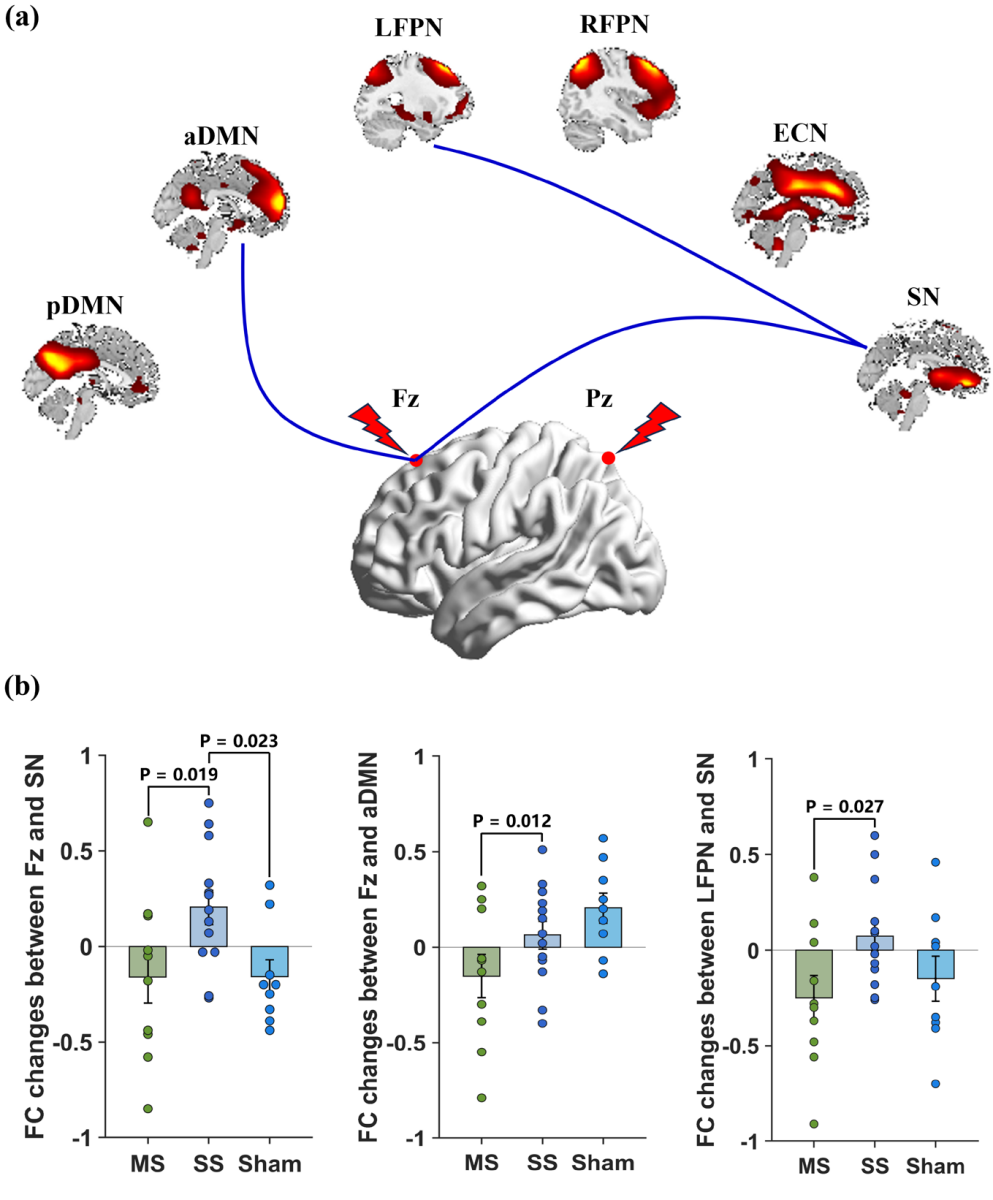
Comparison of the changes of functional connectivity between stimulation sites and RSNs and among RSNs. Compared to single‐site group, functional connectivity between Fz and SN, aDMN, as well as between LFPN and SN decreased more in multi‐site group (a). Blue line indicates greater decrease in multi‐site group. The comparison of these functional connectivity changes among groups was presented in (b). MS, multi‐site; SS, single‐site.

In addition to the functional connectivity between stimulation site and RSNs, the functional connectivity among RSNs was also computed and compared. We found that changes in functional connectivity between LFPN and SN differed significantly between the multi‐site and single‐site groups (*p* = 0.027) (Figure 5).

### 3.6 Correlation Between Functional Connectivity and Cognitive Improvement

**FIGURE 6:**
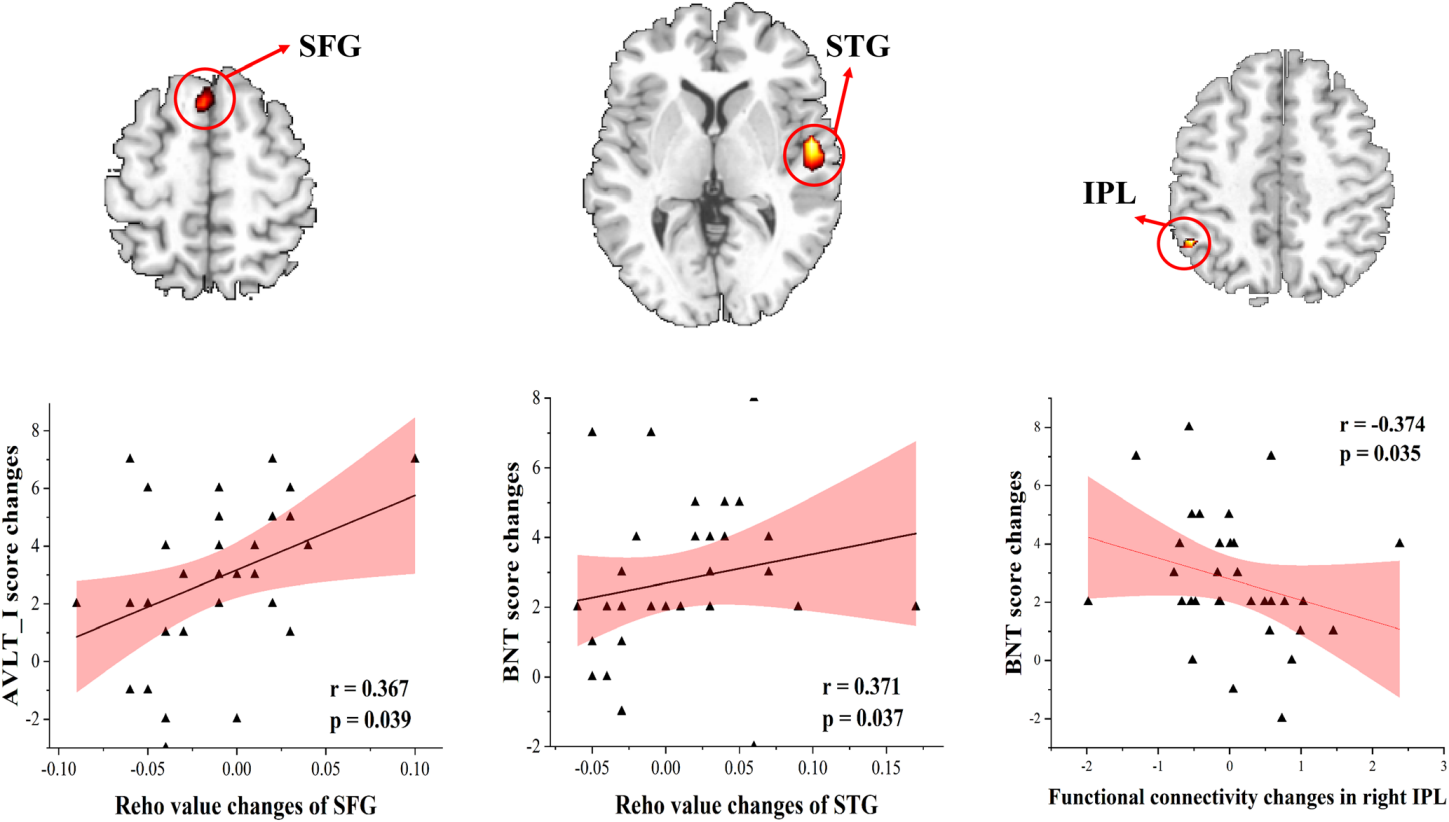
Correlation between the local/remote functional connectivity changes and cognitive score changes. IPL, inferior parietal lobe; SFG, superior frontal gyrus; STG, superior temporal gyrus.

Significant correlations were found between altered Reho values and changes in cognitive scores for the BNT and AVLT‐I. Specifically, the altered Reho value in the left superior frontal gyrus was positively correlated with AVLT‐I score changes after tACS intervention (*r* = 0.367, *p* = 0.039), while altered Reho values in the left superior temporal gyrus were positively correlated with BNT score changes (*r* = 0.371, *p* = 0.036). Furthermore, we observed a significant correlation between altered functional connectivity in the right inferior parietal lobe within the aDMN and BNT score changes (*r* = −0.374, *p* = 0.035) (Figure 6). For the functional connectivity between stimulation sites and RSNs, no significant correlation was found with the cognitive score changes.

## 4 Discussion

We conducted a parallel, placebo‐controlled trial to compare the efficacy of multi‐site and single‐site tACS in improving cognitive functions in patients with MCI. Behavioral results indicated that both multi‐site and single‐site tACS significantly enhanced more domains of cognitive functions compared to sham stimulation, with multi‐site tACS demonstrating superior improvements in language and memory functions. Local and remote functional connectivity analyses revealed that multi‐site tACS induced more extensive modulation of regional functional connectivity and resting‐state functional connectivity, particularly within the DMN, ECN, LFPN, and RFPN. Notably, prefrontal cortex (Fz) stimulation in the multi‐site group further enhanced functional connectivity within and between cognitive networks compared to single‐site tACS. These findings suggest that multi‐site tACS may offer a more comprehensive approach to cognitive enhancement in MCI by modulating multiple brain networks involved in cognitive processing.

### 4.1 Rationale and Justification for the Selection of Stimulation Frequency and Sites

The choice of a 7 Hz stimulation frequency for our tACS protocol was grounded in the central role of θ oscillations in memory and cognitive control. Theta oscillations, first identified in the hippocampus‐entorhinal cortex in animal studies, play an essential role in the temporal organization of working memory items and have been recognized as a key biomarker of working memory [41, 42, 43]. Furthermore, numerous studies have demonstrated abnormal functional connectivity in both θ and α frequency bands in patients with MCI [44, 45]. Our previous research additionally observed significant differences in functional connectivity of θ and α frequency bands between MCI patients and healthy older adults during memory encoding, particularly within the θ band, where MCI patients exhibited more extensive alterations involving broader brain regions compared to the α band [46, 47]. Moreover, a growing body of recent evidence indicates that θ‐tACS can significantly enhance cognitive function in healthy adults [48, 49]. Based on these findings, we selected the θ band as our stimulation. Considering the pathological manifestations in the θ and α bands of MCI patients, we specifically chose 7 Hz—a frequency within the θ range that approaches the α band—as our precise stimulation frequency to potentially maximize therapeutic effects and realize neural modulation.

Regarding the selection of stimulation sites. First, the DLPFC is the most commonly targeted region in clinical practice and research involving brain stimulation for neuropsychiatric disorders, including MCI/AD. Existing meta‐analyses have demonstrated that neuromodulation targeting the DLPFC can significantly improve cognitive function in MCI patients [50, 51]. Second, the DLPFC plays a critical role in various emotional and cognitive processes, including emotion regulation, executive control, and memory processes [52, 53]. Finally, the precuneus is a core brain region implicated in MCI/AD pathogenesis. Previous multimodal neuroimaging studies have consistently identified the precuneus as the area exhibiting the most sever3e and prominent abnormalities in MCI, including Aβ protein deposition, glucose hypometabolism, and aberrant resting‐state brain activity [22, 54, 55]. Consequently, this region is recognized as a pivotal and potentially high‐yield therapeutic target for MCI. Moreover, a circuit‐based rTMS study demonstrated that multi‐target stimulation of the left prefrontal cortex and left precuneus significantly enhances the therapeutic effect of rTMS on long‐term delayed recall memory in patients with MCI [18]. Therefore, the selection of both stimulation frequency and sites in this study is supported by substantial theoretical foundations and demonstrates strong scientific rationale, ensuring the innovation and logical coherence of our research.

### 4.2 Enhanced Cognitive Benefits of Multi‐Site tACS and Combined Interventions

We observed significant improvement across various domains of cognitive functions following 20 days of tACS combined with working memory training, in both multi‐site and single‐site groups. The observed efficacy of tACS in enhancing memory and executive functions aligns with previous meta‐analyses investigating the effects of tACS in healthy adults [49, 56, 57, 58]. These studies reported beneficial effects of tACS on working memory, long‐term memory, declarative memory, executive function, and sensory processes, including sensorimotor, auditory, and visual perception. Furthermore, previous meta‐analyses on tDCS have demonstrated positive effects on global cognition [59], memory [59, 60], language [60], and executive functions [59] in patients with MCI. While studies on tACS in MCI and AD are relatively limited, existing evidence suggests promising efficacy in improving global cognition, memory, and executive functions in these populations [10, 35, 61]. Additionally, a comparative study on the effects of tACS versus tDCS in MCI reported that tACS led to greater improvements in cognitive functions [35]. Therefore, the broad cognitive enhancements observed in our study are consistent with and supported by existing literature. tACS is increasingly regarded as a promising non‐pharmacological intervention, either as a standalone therapy or in combination with pharmacological and/or other behavioral interventions in MCI/ad [11, 62].

Improvements were also observed in the sham group on certain cognitive measures, likely attributable to the placebo effect and the cognitive training component of the intervention. Previous studies have demonstrated that cognitive training and memory‐focused interventions alone can improve cognitive functions in individuals with MCI [63, 64, 65]. However, when cognitive training is combined with other therapeutic approaches, such as non‐invasive brain stimulation [9] or physical exercise [66, 67], significantly greater cognitive benefits are observed compared to single‐modality interventions. tACS, in particular, appears to enhance cognitive functions beyond the effects of cognitive training alone. Moreover, a study investigating the efficacy of multi‐site anodal tDCS in AD patients found that combined tDCS with cognitive stimulation led to significantly greater improvements in cognitive performance compared to sham stimulation [20]. These findings may help explain the superior effects of multi‐site tACS on recognition memory and verbal fluency observed in MCI patients in our study.

### 4.3 Modulation of tACS on Local Functional Connectivity and Cognition‐Related Brain Networks

Previous neuroimaging studies have suggested that AD might be best conceptualized as a disconnection syndrome, characterized by the disrupted structural and functional connectivity across multiple brain regions and networks [14, 15, 16, 17]. Theoretically, effective therapeutic interventions should be able to restore and enhance functional connectivity, particularly within cognition‐related brain networks in patients with MCI. Therefore, we examined local functional connectivity and cognition‐related functional brain networks to explore the underlying neural mechanisms associated with cognitive improvements induced by tACS. ReHo analysis results revealed significant group differences in the superior frontal gyrus and superior temporal gyrus, regions that correspond to the Fz stimulation site and are functionally associated with language processing, respectively. Notably, following tACS treatment, Reho changes in the multi‐site tACS group were primarily observed in the prefrontal cortex, whereas such changes were not evident in the single‐site group or sham group. These findings suggest that multi‐site tACS exerts a stronger modulatory effect on the local functional connectivity, particularly in regions targeted by the Fz stimulation site. This observation aligns with findings from a previous tDCS study, which demonstrated significant increases in Reho values in the frontal lobe, including the middle frontal lobe and superior frontal lobe, following high‐definition tDCS in MCI patients [68]. The prefrontal cortex plays a crucial role in various memory functions, including episodic memory [53, 69], semantic memory [53], replay memory [70], and memory consolidation [52]. Moreover, state‐dependent brain stimulation over the prefrontal cortex has been shown to disrupt memory reconsolidation [71], suggesting that direct stimulation of this region could enhance memory function. Thus, the more pronounced ReHo changes in the multi‐site tACS group likely contribute to its superior cognitive improvements, particularly in memory function, compared to single‐site and sham stimulation. In addition, the Reho changes in the superior frontal cortex and superior temporal gyrus were significantly correlated with improvements in AVLT‐I score (memory function) and BNT scores (language function), respectively. This convergence between neurophysiological and cognitive findings further supports the therapeutic efficacy of multi‐site tACS in enhancing memory and language function in MCI patients.

Currently, only one tDCS study has reported that tDCS enhances the temporal variability of dynamic functional connectivity within DMN, ECN, and SN [30]. Similarly, a gamma‐tACS study observed increased functional connectivity between the hippocampus and inferior parietal lobe, with stronger functional connectivity changes correlating with greater episodic memory improvements [31]. Consistent with these findings, our study observed more extensive functional connectivity changes following multi‐site tACS intervention compared to single‐site and sham stimulation. Specifically, significant functional connectivity alterations were observed primarily within the DMN, ECN, LFPN, as well as increased inter‐functional connectivity between SN and LFPN. These results are consistent with the findings reported in the tDCS [30] and gamma tACS study [31]. Previous neuroimaging studies have confirmed the crucial roles of these networks in MCI. The structural and functional disruptions of these networks have been reported to be associated with MCI‐related cognitive abnormalities [22, 26, 72]. Notably, these networks have also been proposed as critical targets for therapeutic intervention for MCI [27]. Moreover, rTMS has been shown to regulate the aberrant brain activity and functional connectivity within the DMN [19, 73], ECN [74], and FPN [28, 75]. The functional connectivity changes observed in our study suggest that multi‐site tACS similarly modulates cognitive functional networks, providing a potential mechanism for its enhanced cognitive benefits. Additionally, the ECN plays a pivotal role in the working memory processing and the integration of sensory and memory information [76]; while intrinsic LFPN connectivity has been proved to be closely associated with language dysfunction [77, 78]. Therefore, the functional connectivity enhancements within ECN and LFPN after multi‐site tACS are consistent with the greater therapeutic effects observed in memory and language function.

Beyond intra‐functional connectivity within DMN, ECN and LFPN, we also observed significant inter‐functional connectivity difference between LFPN and SN in the multi‐site versus single‐site tACS groups. This may indicate that the superior therapeutic effects of multi‐site tACS on language function may be attributed to stimulation at Fz indirectly modulating the language‐related LFPN via the SN, thereby leading to further improvement in language function. Previous research has demonstrated that structural, functional, and metabolic abnormalities within the SN and FPN can be modulated by repeated high‐definition tDCS [30], cognitive training [79], aerobic training [80], physical exercise [33], and acupuncture [34]. Among these interventions, exercise training has been shown to increase inter‐functional connectivity between the FPN and SN [79], with stronger functional connectivity increases associated with better episodic memory performance. Our findings suggest that multi‐site tACS may exert a similar regulatory effect, potentially explaining its superior impact on memory function. Furthermore, we observed significant differences between the multi‐site and single‐site tACS groups in functional connectivity between the Fz stimulation site and SN, as well as between the Fz stimulation site and DMN. These results highlight that increased tACS stimulation over the frontal cortex in the multi‐site condition may enhance its modulatory effects on memory and language functions by directly influencing SN and DMN connectivity. The Fz and Pz electrode placements in the multi‐site tACS group targeted the prefrontal and parietal cortices, which are key nodes in the DMN, ECN, and FPN [81, 82, 83]. By sequentially stimulating these regions, multi‐site tACS may enhance integration and communication between these cognitive networks, leading to broader cognitive improvements. The finding that multi‐site tACS specifically strengthened functional connectivity between the Fz site and the SN and DMN, as well as between LFPN and SN, further supports this interpretation. The SN plays a crucial role in detecting and integrating salient stimuli and is responsible for dynamically switching between the DMN and ECN [84, 85, 86]. Enhanced functional connectivity between the SN and these networks may facilitate more efficient cognitive control and information processing, contributing to the observed language and memory function improvements.

### 4.4 Limitations

While our findings support the superior effects of multi‐site tACS on improving cognitive functions in MCI, several limitations should be acknowledged: (1) Small sample size—the relatively small sample may limit the generalizability of our results. Especially in MRI data analysis, fewer MCI patients are enrolled because interventional studies require both pre‐ and post‐treatment MRI images to meet quality standards. Future large‐scale, multicenter studies are needed to confirm these findings and examine individual variability in response to tACS. (2) Lack of long‐term follow‐up—this study did not assess the long‐term maintenance of cognitive improvements induced by tACS. Future research should evaluate the durability of tACS effects and its potential role in delaying MCI‐to‐AD progression. (3) MCI subtype analysis—although most participants had amnestic MCI, we did not conduct comparative analyses between amnestic and non‐amnestic MCI subtypes. Future studies should explore potential differential responses to tACS across MCI subtypes. Therefore, future studies with large sample sizes and specific disease subtypes are needed to validate the finding observed in our study.

## 5 Conclusions

This study provides strong evidence that multi‐site tACS is more effective than single‐site tACS in enhancing cognitive functions and modulating brain networks in MCI patients. Multi‐site tACS exerts superior neuromodulatory effects through dual‐phase stimulation of prefrontal‐precuneus circuits to enhance working memory consolidation and language fluency. Furthermore, by improving functional connectivity within and between key cognitive networks, multi‐site tACS represents a promising non‐pharmacological intervention for MCI. Future research should optimize tACS protocols and investigate its long‐term efficacy in mitigating cognitive decline in MCI and AD.

## Author Contributions

Z.G. and N.J. designed the study. Z.G. and Y.J. collected the data. Z.G., Y.J., and J.H. performed and interpreted statistical data analysis, fMRI imaging processing, and prepared the figures. Z.G. and N.J. wrote the main manuscript text. All authors reviewed the manuscript.

## Funding

This research was supported by the National Clinical Research Center for Geriatrics, West China Hospital, Sichuan University (Grant No. Z2024YY002), the Fundamental Research Funds for the Central Universities (Grant No. YJ202373), the Key Research Project Grant from the National Clinical Research Center for Geriatrics under Grant (Grant No. Z2023YY001), the Bureau of Science and Technology Nanchong City (Grant No. 22SXQT0345), and the Sichuan Medical and Health Care Promotion Institute Scientific Research Project (Grant No. KY2024SJ0055).

## Ethics Statement

This study was reviewed and approved by the Ethics Committee of the Second Clinical Medical College of North Sichuan Medical College [Approval ID: 2024 (065)] and the Ethics Committee on Biomedical Research, West China Hospital of Sichuan University [Approval ID: 2024 (1422)]. This study was conducted in full accordance with the ethical principles for medical research involving human subjects as outlined in the Declaration of Helsinki.

## Consent

Prior to participation, written informed consent was obtained from all individual participants included in the study. The consent process ensured that participants were fully informed about the study's purpose, procedures, potential risks and benefits, and their right to withdraw at any time without penalty.

## Conflicts of Interest

The authors declare no conflicts of interest.

## Supporting information


**Data S1:** cns70707‐sup‐0001‐DataS1.docx.

## Data Availability

The data that support the findings of this study are available from the corresponding author upon reasonable request.
