# Supplementary material for: Multi‐Site Theta‐tACS Improves Memory and Language Performance and Associated Local and Remote Functional Connectivity in Mild Cognitive Impairment
Source: CNS Neurosci Ther. 2025 Dec 18;31(12):e70707. doi: 10.1002/cns.70707 (PMC12715273; doi:10.1002/cns.70707)
Supplement: Supplementary file 1 — Data S1: cns70707‐sup‐0001‐DataS1.docx. [file CNS-31-e70707-s001.docx]

**Supplementary Materials**

In our study, the comparisons of the Reho and intra- functional connectivity of each RSN were all conducted among multi-site, single-site, and sham groups. Besides, the comparisons of the Reho and intra- functional connectivity of each RSN were also performed between baseline and post-treatment in each group. Information the brain regions showing significant differences in the comparison were presented in Table s1 and Table s2, which provided significant results of Reho and functional connectivity, respectively.

Table s1 Brain regions showing significant Reho differences among groups

| Brain Region | Side | Peak Coordinate | Peak Intensity | Cluster Size |
| --- | --- | --- | --- | --- |
| **ANOVA Group Effects** | | | | |
| Superior Temporal Gyrus | Left | -54, -6, 0 | 19.57 | 69 |
| Superior Frontal Gyrus | Bilateral | 3, 18, 57 | 15.31 | 47 |
| **Post-tACS vs Baseline (Multi-site Group)** | | | | |
| Middle Frontal Gyrus | Right | 33, -3, 54 | 7.52 | 16 |
| Supplementary Motor Area | Left | -12, -12, 63 | -6.11 | 29 |
| **Post-tACS vs Baseline (Single-site Group)** | | | | |
| Inferior Occipital Lobe | Left | -21, -93, -3 | 4.30 | 36 |

tACS, transcranial alternating current stimulation.

Table s2 Brain Regions showing significant functional connectivity differences among groups

| RSN | Brain Region | Side | Peak Coordinate | Peak Intensity | Cluster Size |
| --- | --- | --- | --- | --- | --- |
| **All groups comparison** | | | | | |
| **aDMN** | Superior Frontal Gyrus | Right | 24, 54, 33 | 8.19 | 39 |
| **pDMN** | Precuneus | Left | -9, -69, 33 | 10.04 | 26 |
|  | Inferior Parietal Lobe | Right | 48, -54, 45 | 10.39 | 14 |
| **ECN** | Middle Frontal Gyrus | Left | -30, 45, 24 | 14.98 | 50 |
| **LFPN** | Inferior Parietal Lobe | Left | -57, -30, 45 | 9.02 | 13 |
| **RFPN** | Middle Frontal Gyrus | Right | 42, 6, 51 | 11.74 | 33 |
| **Post-tACS vs Baseline** | | | | | |
| **Multi-site group** | | | | | |
| **aDMN** | Precuneus | Right | 12, -45, 51 | -9.31 | 50 |
|  | Middle Frontal Gyrus | Left | -48, 30, 33 | -5.85 | 62 |
|  | Superior Frontal Gyrus | Right | 24, -9, 69 | 4.90 | 42 |
| **pDMN** | Hippocampus | Right | 39, -12, -18 | -4.85 | 42 |
|  | Parahippocampal Gyrus | Left | -20, -34, -14 | -5.60 | 32 |
|  | Inferior Parietal Lobe | Right | 33, -45, 45 | 5.91 | 28 |
| **ECN** | Superior Medial Frontal Gyrus | Left | -6, 51, 3 | 5.85 | 54 |
|  | Superior Parietal Lobe | Left | -39, -63, 54 | 5.78 | 19 |
| **LFPN** | Superior Frontal Gyrus | Left | -21, 24, 39 | 6.50 | 58 |
| **Single-site Group** | | | | | |
| **aDMN** | Precuneus | Right | 18, -57, 21 | 3.66 | 16 |
|  | Inferior Parietal Lobe | Left | -51, -39, 36 | -3.79 | 13 |
|  | Superior Temporal Gyrus | Right | 54, -21, 12 | 6.58 | 26 |
| **Sham Group** | | | | | |
| **aDMN** | Inferior Parietal Lobe | Left | -42, -54, 48 | -10.15 | 60 |

tACS, transcranial alternating current stimulation; RSN, resting-state network; aDMN, anterior default mode network; pDMN, posterior default mode network; ECN, executive control network; LFPN, left frontoparietal network; RFPN, right frontoparietal network.
